# Privacy-protecting estimation of adjusted risk ratios using modified Poisson regression in multi-center studies

**DOI:** 10.1186/s12874-019-0878-6

**Published:** 2019-12-05

**Authors:** Di Shu, Jessica G. Young, Sengwee Toh

**Affiliations:** Department of Population Medicine, Harvard Medical School and Harvard Pilgrim Health Care Institute, Boston, MA USA

**Keywords:** Distributed analysis, Modified Poisson regression, Multi-center studies, Odds ratio, Privacy protection, Risk ratio

## Abstract

**Background:**

Multi-center studies can generate robust and generalizable evidence, but privacy considerations and legal restrictions often make it challenging or impossible to pool individual-level data across data-contributing sites. With binary outcomes, privacy-protecting distributed algorithms to conduct logistic regression analyses have been developed. However, the risk ratio often provides a more transparent interpretation of the exposure-outcome association than the odds ratio. Modified Poisson regression has been proposed to directly estimate adjusted risk ratios and produce confidence intervals with the correct nominal coverage when individual-level data are available. There are currently no distributed regression algorithms to estimate adjusted risk ratios while avoiding pooling of individual-level data in multi-center studies.

**Methods:**

By leveraging the Newton-Raphson procedure, we adapted the modified Poisson regression method to estimate multivariable-adjusted risk ratios using only summary-level information in multi-center studies. We developed and tested the proposed method using both simulated and real-world data examples. We compared its results with the results from the corresponding pooled individual-level data analysis.

**Results:**

Our proposed method produced the same adjusted risk ratio estimates and standard errors as the corresponding pooled individual-level data analysis without pooling individual-level data across data-contributing sites.

**Conclusions:**

We developed and validated a distributed modified Poisson regression algorithm for valid and privacy-protecting estimation of adjusted risk ratios and confidence intervals in multi-center studies. This method allows computation of a more interpretable measure of association for binary outcomes, along with valid construction of confidence intervals, without sharing of individual-level data.

## Background

In studies where the outcome variable is binary, a logistic regression model is commonly used for convenient estimation of the adjusted (i.e., conditional on measured covariates) odds ratio comparing exposed to unexposed individuals [1, 2]. However, the odds ratio is not easily interpretable because, unlike the risk ratio, it is not a direct measure of a ratio of probabilities, often of primary interest to patients and clinicians [3]. Although the odds ratio approximates the risk ratio under the rare disease assumption (e.g., odds of the outcome < 10% in all exposure and confounder categories) [4], it can be quite different from the risk ratio and produce misleading results when this assumption is not met [3, 5–8].

Log-binomial regression can directly estimate adjusted risk ratios without requiring the rare disease assumption, but it is susceptible to non-convergence issues when the maximum likelihood estimators lie near the boundary of the parameter space [9, 10]. Poisson regression is another approach to estimating adjusted risk ratios and does not have any known convergence problems in its parameter space. This approach provides consistent estimates of adjusted risk ratios but incorrect estimates of the variance because it relies on a Poisson distributed, rather than binomially distributed, outcome. In practice, standard implementation of Poisson regression tends to produce conservative confidence intervals [11]. As a solution to these challenges, Zou [12] proposed a modified Poisson regression approach that allows direct estimation of adjusted risk ratios even when the rare disease assumption is not met. This approach avoids the convergence issues typically observed in log-binomial regression and, unlike conventional Poisson regression, provides consistent variance estimates and confidence intervals with the correct nominal coverage.

A growing number of studies are now conducted within multi-center distributed data networks [13]. Such collaborations combine data from multiple sources to generate more reliable evidence using larger and more representative samples. Within these networks, each data-contributing site (i.e., data partner) maintains physical control of their data and may not always be able or willing to share individual-level data for analysis. For example, the Sentinel System is a national program funded by the U.S. Food and Drug Administration to proactively monitor the safety of regulated medical products using electronic healthcare data from multiple data partners [14]. In multi-center studies like those conducted within the Sentinel System, it is often crucial to minimize sharing of sensitive individual-level data to protect patient privacy. The development and applications of analytic methods that enable valid statistical analysis without pooling individual-level data are therefore increasingly important.

Privacy-protecting distributed algorithms to conduct logistic regression analyses have been previously developed [15–18]. To our knowledge, there are currently no distributed algorithms to estimate adjusted risk ratios via modified Poisson regression while avoiding pooling of individual-level data across data partners. In this paper, we propose such an algorithm and provide example R [19] code to implement the algorithm. We also illustrate in simulated and real-world data examples that our algorithm produces adjusted risk ratio estimates and standard errors equivalent to those obtained from the corresponding pooled individual-level data analysis.

## Methods

### Theory of modified Poisson regression for pooled individual-level data

We begin by describing the general theory of modified Poisson regression in single-database studies. Let ***X*** be a vector of covariates, *E* a binary exposure indicator (*E* = 1 if exposed and *E* = 0 if unexposed), and *Y* the binary outcome variable (*Y* = 1 if the outcome occurs and *Y* = 0 otherwise).

Let ***Z*** be a vector of information on the exposure and covariates. Specifically, ***Z =*** (1, ***g***(*E*, ***X***^*T*^))^*T*^ where ***g***(*E*, ***X***^*T*^) is a vector containing a specified function of *E* and ***X***. Assume the risk of the outcome conditional on *E* and ***X*** can be written as

1 $$ P\left(Y=1|E,\boldsymbol{X}\right)=\exp \left({\boldsymbol{\beta}}^T\boldsymbol{Z}\right) $$

where ***β*** is an unknown vector of parameters. An example of model 1 is $P\left(Y=1|E,\boldsymbol{X}\right)=\exp \left({\beta}_0+{\beta}_E\ E+{\boldsymbol{\beta}}_{\boldsymbol{X}}^T\boldsymbol{X}\right)$. In this special case, the risk ratio of the outcome comparing the exposed to unexposed and adjusting for covariates ***X*** is given by *P*(*Y* = 1| *E* = 1, ***X***)/*P*(*Y* = 1| *E* = 0, ***X***) = exp(*β*_*E*_).

Alternatively, we might assume a more flexible model that allows interactions between *E* and ***X***: $P\left(Y=1|\boldsymbol{X},E\right)=\exp \left({\beta}_0+{\beta}_E\ E+{\boldsymbol{\beta}}_{\boldsymbol{X}}^T\boldsymbol{X}+{\boldsymbol{\beta}}_{E\boldsymbol{X}}^TE\boldsymbol{X}\right)$. Under this model, the risk ratio of the outcome comparing the exposed to unexposed and adjusting for covariates ***X*** is given by $P\left(Y=1|E=1,\boldsymbol{X}\right)/P\left(Y=1|E=0,\boldsymbol{X}\right)=\exp \left({\beta}_E+{\boldsymbol{\beta}}_{E\boldsymbol{X}}^T\boldsymbol{X}\right)$, which depends on the value of ***X***.

Suppose we have an independent and identically distributed sample of size *n*. For each individual *i*, the following variables are measured: Let ***X***_*i*_ be a vector of covariates, *E*_*i*_ a binary exposure indicator (*E*_*i*_ = 1 if exposed and *E*_*i*_ = 0 if unexposed), *Y*_*i*_ the binary outcome variable (*Y*_*i*_ = 1 if the outcome occurs and *Y*_*i*_ = 0 otherwise), and ***Z***_*i*_ a vector of information on the exposure and covariates, i.e., ***Z***_*i*_ ***=*** (1, ***g***(*E*_*i*_, ***X***_*i*_^*T*^))^*T*^**.**

Zou [12] provided the theoretical justification for his proposed approach in the setting of a 2 by 2 table (a binary exposure and no covariates). The justification for this approach can be established more generally using the theory of unbiased estimating equations [20]. Provided model 1 is correctly specified, we have *E*[{*Y* − exp(***Z***^*T*^***β***)}***Z***] = **0**, which leads to the unbiased estimating equation

2 $$ \sum \limits_{i=1}^n\left\{{Y}_i-\exp \left({{\boldsymbol{Z}}_i}^T\boldsymbol{\beta} \right)\right\}{\boldsymbol{Z}}_i=\mathbf{0} $$

Solving (2) for ***β*** gives $\hat{\boldsymbol{\beta}}$, a consistent and asymptotically normal estimator for the true ***β***.

A consistent estimator of the variance of $\hat{\boldsymbol{\beta}}$ is then given by the sandwich variance estimator [20]

3 $$ \hat{\mathit{\operatorname{var}}}\left(\hat{\boldsymbol{\beta}}\right)=\left\{\boldsymbol{H}{\left(\hat{\boldsymbol{\beta}}\right)}^{-1}\right\}\boldsymbol{B}\left(\hat{\boldsymbol{\beta}}\right)\left\{\boldsymbol{H}{\left(\hat{\boldsymbol{\beta}}\right)}^{-1}\right\} $$

where $\boldsymbol{H}\left(\hat{\boldsymbol{\beta}}\right)=-\sum \limits_{i=1}^n\exp \left({{\boldsymbol{Z}}_i}^T\hat{\boldsymbol{\beta}}\right){\boldsymbol{Z}}_i{{\boldsymbol{Z}}_i}^T$ and $\boldsymbol{B}\left(\hat{\boldsymbol{\beta}}\right)=\sum \limits_{i=1}^n{\left\{{Y}_i-\exp \left({{\boldsymbol{Z}}_i}^T\hat{\boldsymbol{\beta}}\right)\right\}}^2{\boldsymbol{Z}}_i{{\boldsymbol{Z}}_i}^T$. Zou [12] referred to this procedure as *modified Poisson regression* because (2) is equivalent to the score equation for the Poisson likelihood but the variance estimator does not rely on the Poisson distribution assumption (clearly unreasonable for binary outcomes).

### Distributed algorithm for conducting modified Poisson regression in multi-center studies

Suppose the *n* individuals’ data are physically stored in *K* data partners that are unable to share their individual-level data with the analysis center. For *k* = 1, …, *K*, let *Ω*_*k*_ denote the set of indexes of individuals who are members of the *k* ^th^ data partner.

When the individual-level data are available to the analysis center, the estimator $\hat{\boldsymbol{\beta}}$ and its corresponding variance estimator can be obtained with off-the-shelf statistical software. However, when the individual-level data are not available, (2) cannot be directly solved for ***β*** to obtain $\hat{\boldsymbol{\beta}}$, and $\hat{\mathit{\operatorname{var}}}\left(\hat{\boldsymbol{\beta}}\right)$ cannot be directly calculated using (3). Here we describe a distributed algorithm that produces identical $\hat{\boldsymbol{\beta}}$ and $\hat{\mathit{\operatorname{var}}}\left(\hat{\boldsymbol{\beta}}\right)$ in multi-center studies where individual-level data are not pooled.

We leverage the Newton-Raphson method such that $\hat{\boldsymbol{\beta}}$ can be obtained using an iteration-based procedure with only summary-level information being shared between the data partners and the analysis center in each iteration. The *r* ^th^ iterated estimate of ***β*** using the Newton-Raphson method is

4 $$ {\boldsymbol{\beta}}^{\left(\mathrm{r}\right)}={\boldsymbol{\beta}}^{\left(\mathrm{r}-1\right)}-{\left\{{\boldsymbol{H}}^{(r)}\right\}}^{-1}{\boldsymbol{S}}^{(r)} $$

where ***β***^(r − 1)^ is the (*r* − 1) ^th^ iterated estimate of ***β***, ${\boldsymbol{S}}^{(r)}=\sum \limits_{i=1}^n\left\{{Y}_i-\exp \left({{\boldsymbol{Z}}_i}^T{\boldsymbol{\beta}}^{\left(\mathrm{r}-1\right)}\right)\right\}{\boldsymbol{Z}}_i$, and ${\boldsymbol{H}}^{(r)}=-\sum \limits_{i=1}^n\exp \left({{\boldsymbol{Z}}_i}^T{\boldsymbol{\beta}}^{\left(\mathrm{r}-1\right)}\right){\boldsymbol{Z}}_i{{\boldsymbol{Z}}_i}^T.$

We observe that ***S***^(*r*)^ and ***H***^(*r*)^ can be re-written as summation of site-specific quantities:

5 $$ {\boldsymbol{S}}^{(r)}=\sum \limits_{k=1}^K{{\boldsymbol{S}}_k}^{(r)} $$

where

6 $$ {{\boldsymbol{S}}_k}^{(r)}=\sum \limits_{i\text{\EUR} {\varOmega}_k}\left\{{Y}_i-\exp \left({{\boldsymbol{Z}}_i}^T{\boldsymbol{\beta}}^{\left(\mathrm{r}-1\right)}\right)\right\}{\boldsymbol{Z}}_i $$

and

7 $$ {\boldsymbol{H}}^{(r)}=\sum \limits_{k=1}^K{{\boldsymbol{H}}_k}^{(r)} $$

where

8 $$ {{\boldsymbol{H}}_k}^{(r)}=-\sum \limits_{i\text{\EUR} {\varOmega}_k}\exp \left({{\boldsymbol{Z}}_i}^T{\boldsymbol{\beta}}^{\left(\mathrm{r}-1\right)}\right){\boldsymbol{Z}}_i{{\boldsymbol{Z}}_i}^T $$

Therefore, to calculate ***S***^(*r*)^ and ***H***^(*r*)^, each data partner *k* = 1, …, *K* only needs to calculate and share with the analysis center the summary-level information ***S***_*k*_^(*r*)^ and ***H***_*k*_^(*r*)^.

Next, consider estimation of the variance of $\hat{\boldsymbol{\beta}}$. We observe that

9 $$ \boldsymbol{H}\left(\hat{\boldsymbol{\beta}}\right)=\sum \limits_{k=1}^K{\boldsymbol{H}}_k\left(\hat{\boldsymbol{\beta}}\right) $$

where

10 $$ {\boldsymbol{H}}_k\left(\hat{\boldsymbol{\beta}}\right)=-\sum \limits_{i\text{\EUR} {\varOmega}_k}\exp \left({{\boldsymbol{Z}}_i}^T\hat{\boldsymbol{\beta}}\right){\boldsymbol{Z}}_i{{\boldsymbol{Z}}_i}^T $$

and

11 $$ \boldsymbol{B}\left(\hat{\boldsymbol{\beta}}\right)=\sum \limits_{k=1}^K{\boldsymbol{B}}_k\left(\hat{\boldsymbol{\beta}}\right) $$

where

12 $$ {\boldsymbol{B}}_k\left(\hat{\boldsymbol{\beta}}\right)=\sum \limits_{i\text{\EUR} {\varOmega}_k}{\left\{{Y}_i-\exp \left({{\boldsymbol{Z}}_i}^T\hat{\boldsymbol{\beta}}\right)\right\}}^2{\boldsymbol{Z}}_i{{\boldsymbol{Z}}_i}^T $$

To calculate $\boldsymbol{H}\left(\hat{\boldsymbol{\beta}}\right)$ and $\boldsymbol{B}\left(\hat{\boldsymbol{\beta}}\right)$, each data partner *k* = 1, …, *K* only needs to calculate and share the summary-level information ${\boldsymbol{H}}_k\left(\hat{\boldsymbol{\beta}}\right)$ and ${\boldsymbol{B}}_k\left(\hat{\boldsymbol{\beta}}\right)$ after receiving the value of $\hat{\boldsymbol{\beta}}$ from the analysis center. Unlike in the estimation of ***β***, no iterations are needed in the sandwich variance estimation.

We summarize our distributed algorithm for conducting modified Poisson regression in multi-center studies below.

#### Point estimation

##### Step 0 (Determination of starting values)

The analysis center specifies the starting values for the components of ***β***^(0)^ and sends these values to all data partners. Then, for each iteration *r* until the convergence criteria are met, the following two steps are repeated:

##### Step 1 (*r*^*th*^ iteration of data partners)

Each data partner *k* = 1, …, *K* calculates ***S***_*k*_^(*r*)^ and ***H***_*k*_^(*r*)^ using (6) and (8), respectively, based on ***β***^(r − 1)^ received from the analysis center. All data partners then share the values of ***S***_*k*_^(*r*)^ and ***H***_*k*_^(*r*)^ with the analysis center.

##### Step 2 (*r*^*th*^ iteration of the analysis center)

The analysis center calculates ***S***^(*r*)^ and ***H***^(*r*)^ using (5) and (7), respectively. The analysis center then calculates ***β***^(r)^ using (4) and shares the value of ***β***^(r)^ with all data partners.

The iteration procedure is considered to have converged when the change in the estimates between iterations is within a user-specified tolerance value. In numerical studies to be presented later, we considered a convergence criterion to be met at the (*R* + 1) ^th^ iteration if $\underset{l}{\max}\left|{\delta_l}^{\left(\mathrm{R}+1\right)}\right|<{10}^{-8}$, where *δ*_*l*_^(R + 1)^ = *β*_*l*_^(R + 1)^ − *β*_*l*_^(R)^ if ∣*β*_*l*_^(R)^ ∣  < 0.01 and *δ*_*l*_^(R + 1)^ ***=*** (*β*_*l*_^(R + 1)^ − *β*_*l*_^(R)^)/*β*_*l*_^(R)^ otherwise, and *β*_*l*_^(R)^ is the *l* ^th^ element of ***β***^(R)^. Once achieving convergence, the analysis center shares the final estimate $\hat{\boldsymbol{\beta}}$ with all data partners.

#### Variance estimation

##### Step 1 (Calculation of summary-level information by data partners)

Each data partner *k* = 1, …, *K* calculates ${\boldsymbol{H}}_k\left(\hat{\boldsymbol{\beta}}\right)$ and ${\boldsymbol{B}}_k\left(\hat{\boldsymbol{\beta}}\right)$ using (10) and (12), respectively, and then shares the values of ${\boldsymbol{H}}_k\left(\hat{\boldsymbol{\beta}}\right)$ and ${\boldsymbol{B}}_k\left(\hat{\boldsymbol{\beta}}\right)$ with the analysis center.

##### Step 2 (Calculation of the variance estimate by the analysis center)

The analysis center calculates $\boldsymbol{H}\left(\hat{\boldsymbol{\beta}}\right)$ and $\boldsymbol{B}\left(\hat{\boldsymbol{\beta}}\right)$ using (9) and (11), respectively, and then calculates the estimated variance $\hat{\mathit{\operatorname{var}}}\left(\hat{\boldsymbol{\beta}}\right)$ using (3).

Due to mathematical equivalence, the above procedure would provide the same point estimates and sandwich variance estimates as the analysis that uses individual-level data pooled across data partners.

## Results

### Analysis of simulated data

We considered a simulation design that enabled us to assess the performance of the proposed summary-level modified Poisson method in the presence of multiple data partners, multiple covariates (including but not limited to data source indicators), and differences in exposure prevalence and outcome incidence across data partners. Although modified Poisson regression is broadly applicable with rare and common outcomes, here we considered a scenario with common outcomes, where logistic regression would provide biased estimates of adjusted risk ratios.

Specifically, we simulated a distributed network with three (i.e., *K* = 3) data partners and *n* = 10000 individuals with 5000, 2000, and 3000 individuals contributing data from the first, second, and third data partners, respectively. We considered five covariates *X*_1_, *X*_2_, *X*_3_, *X*_4_ and *X*_5_. We generated *X*_1_ as a Bernoulli variable with a mean (i.e., *P*(*X*_1_ = 1)) of 0.6, *X*_2_ as a continuous variable following the standard uniform distribution, *X*_3_ as a continuous variable following the unit exponential distribution, *X*_4_ as an indicator that an individual contributed data from the first data partner, and *X*_5_ as an indicator that an individual contributed data from the second data partner.

The exposure *E* was generated from a Bernoulli distribution with the probability of being exposed (*E* = 1) defined as 1/{1 + exp(0.73 − *X*_1_ − *X*_2_ + *X*_3_ − 0.2*X*_4_ + 0.2*X*_5_)}, indicating a non-randomized study. This setting led to different exposure prevalences across data partners. The resulting exposure prevalence was approximately 40% overall, 43% for the first data partner, 34% for the second data partner, and 38% for the third data partner. The outcome *Y* was generated from a Bernoulli distribution with the probability of having the outcome (*Y* = 1) defined as exp(***Z***^*T*^***β***) = exp(−0.1 − 0.5*E* − 0.4*X*_1_ − 0.6*X*_2_ − 0.5*X*_3_ − 0.1*X*_4_ + 0.1*X*_5_) such that the true adjusted risk ratio comparing the exposed to unexposed was exp(−0.5) = 0.61. The resulting outcome incidence (i.e., risk) varied across the three data partners. This incidence was about 30% for the entire pooled data, 27% for the first data partner, 35% for the second data partner, and 31% for the third data partner.

As the reference, we first fit a modified Poisson regression model using pooled individual-level data (Table 1). We then implemented our proposed distributed algorithm that did not require sharing of individual-level data to estimate ***β***. Based on the starting value ***β***^(0)^ = **0**, the analysis took seven iterations to converge. The individual-level and summary-level methods produced identical point estimates and sandwich variance-based standard errors (Table 1). The Additional file 1 provides the summary-level information shared between the data partners and the analysis center during each iteration.

**Table 1:** Point Estimates and Standard Errors Using the Summary-Level Modified Poisson Method and Pooled Individual-Level Data Analysis: Analysis of Simulated Data

Covariates	Summary-Level Modified Poisson Method		Pooled Individual-Level Data Analysis	
	Parameter Estimates	Standard Errors	Parameter Estimates	Standard Errors
Intercept	−0.09702882	0.03751991	−0.09702882	0.03751991
Exposure	−0.48776703	0.03462485	−0.48776703	0.03462485
*X*_1_	−0.38249121	0.02968448	−0.38249121	0.02968448
*X*_2_	−0.62968463	0.05161073	−0.62968463	0.05161073
*X*_3_	−0.50382664	0.02389781	−0.50382664	0.02389781
*X*_4_	−0.09079213	0.03389126	−0.09079213	0.03389126
*X*_5_	0.13274741	0.03814272	0.13274741	0.03814272

### Analysis of real-world data

To further illustrate our method, we analyzed a dataset created from the IBM® Health MarketScan® Research Databases, which contain de-identified individual-level healthcare claims information from employers, health plans, hospitals, and Medicare and Medicaid programs fully compliant with U.S. privacy laws and regulations (e.g., Health Insurance Portability and Accountability Act). The study dataset included 9736 patients aged 18–79 years who received sleeve gastrectomy or Roux-en-Y gastric bypass between 1/1/2010 and 9/30/2015. The outcome of interest was any hospitalization during the 2-year follow-up period after surgery. The exposure variable was set to 1 if the patient received sleeve gastrectomy and 0 if the patient received Roux-en-Y gastric bypass. We estimated the risk ratio of hospitalization comparing sleeve gastrectomy with Roux-en-Y gastric bypass using the pooled individual-level data analysis and the summary-level information approach, adjusting for the following covariates identified during the 365-day period prior to the surgery: age; sex; Charlson/Elixhauser combined comorbidity score; diagnosis of asthma, atrial fibrillation, atrial flutter, coronary artery disease, deep vein thrombosis, gastroesophageal reflux disease, hypertension, ischemic stroke, myocardial infarction, pulmonary embolism, and sleep apnea; use of anticoagulants, assistive walking device, and home oxygen; unique drug classes dispensed and unique generic medications dispensed.

Of the 9736 patients in the study dataset, 7877 (81%) patients underwent the sleeve gastrectomy procedure and 1859 (19%) patients had the Roux-en-Y gastric bypass procedure. The outcome event was not rare in the study, with 1485 (19%) sleeve gastrectomy patients and 608 (33%) Roux-en-Y gastric bypass patients having at least one hospitalization during the two-year follow-up period. We randomly partitioned the dataset into three smaller datasets with 2000, 3000 and 4736 patients to create a “simulated” distributed data network. As the reference, the pooled individual-level data analysis produced ${\hat{\beta}}_E=-0.4632219$ with a standard error 0.0422368, and a 95% confidence interval: − 0.5460061, − 0.3804377. These results corresponded to an adjusted risk ratio of $\exp \left({\hat{\beta}}_E\right)=0.63$ with a 95% confidence interval: 0.58, 0.68. Based on the starting value ***β***^(0)^ = **0**, the proposed summary-level modified Poisson method took seven iterations to converge and produced point estimates and sandwich variance-based standard errors identical to those observed in the corresponding pooled individual-level data analysis (Table 2). The adjusted odds ratio from logistic regression was 0.53. As expected, interpreting the estimated adjusted odds ratio as an estimate of the adjusted risk ratio amplified the protective effect of sleeve gastrectomy compared to Roux-en-Y gastric bypass, resulting in an effect estimate that was further from the null (suggesting a 10% greater relative protective effect) than the modified Poisson regression estimate.

**Table 2:** Point Estimates and Standard Errors Using the Summary-Level Modified Poisson Method and Pooled Individual-Level Data Analysis*:* Analysis of Real-World Data

Covariates	Summary-Level Modified Poisson Method		Pooled Individual-Level Data Analysis	
	Parameter Estimates	Standard Errors	Parameter Estimates	Standard Errors
Intercept	−1.5653147	0.1040146	−1.5653147	0.1040146
Exposure^a^	−0.4632219	0.0422368	−0.4632219	0.0422368
Demographics				
Age (years)	0.0029253	0.0018694	0.0029253	0.0018694
Female sex	0.0860355	0.0478638	0.0860355	0.0478638
Combined comorbidity score	0.0543412	0.0125169	0.0543412	0.0125169
Diagnosis of				
Asthma	−0.0212109	0.0538624	−0.0212109	0.0538624
Atrial fibrillation	0.0835820	0.1249359	0.0835820	0.1249359
Atrial flutter	0.1638268	0.2598721	0.1638268	0.2598721
Coronary artery disease	0.1597157	0.0746721	0.1597157	0.0746721
Deep vein thrombosis	0.1738365	0.1371837	0.1738365	0.1371837
Gastroesophageal reflux disease	−0.0586971	0.0393428	−0.0586971	0.0393428
Hypertension	0.0046885	0.0448018	0.0046885	0.0448018
Ischemic stroke	−0.1592596	0.1986055	−0.1592596	0.1986055
Myocardial infarction	0.2069423	0.1509918	0.2069423	0.1509918
Pulmonary embolism	0.2846648	0.1436404	0.2846648	0.1436404
Sleep apnea	−0.0261550	0.0396516	−0.0261550	0.0396516
Use of				
Anticoagulants	0.1064905	0.1205252	0.1064905	0.1205252
Assistive walking device	0.0908909	0.1394227	0.0908909	0.1394227
Home oxygen	0.0732620	0.1162295	0.0732620	0.1162295
Number of drug dispensing				
Unique drug classes	−0.0579458	0.0166577	−0.0579458	0.0166577
Unique generic medications	0.0673783	0.0136406	0.0673783	0.0136406

^a^sleeve gastrectomy vs. Roux-en-Y gastric bypass

As expected, we had difficulty fitting a log-binomial regression model within this bariatric surgery dataset. Under the starting value ***β***^(0)^ = **0**, the first iterated estimate ***β***^(1)^ could not be calculated because the formula for ***β***^(1)^ under the log-binomial regression model includes 1 − exp(***Z***_*i*_^*T*^***β***^(0)^) in the denominator, which takes the value 0 when ***β***^(0)^ = **0**. We also considered three non-zero starting values. We first set the starting values for all parameters to 0.05, but the analysis stopped at the second iteration due to matrix singularity. We then let the starting values be the estimates obtained from a logistic regression model fit using the entire bariatric surgery dataset, and the analysis converged with ${\hat{\beta}}_E=-0.7369683$. Finally, we specified the starting values as the estimates obtained from the modified Poisson regression fit using the entire bariatric surgery dataset, and the analysis converged with ${\hat{\beta}}_E=-0.4544135$. These results illustrated the convergence problems of log-binomial regression and the sensitivity of this method to starting values. In comparison, the modified Poisson analysis had no convergence problems and its estimates remained the same as those presented in Table 2 when using these alternative starting values.

## Discussion

In this paper, we proposed and demonstrated – in both simulated and real-world data – a method that adapts the modified Poisson approach to directly estimate adjusted risk ratios in multi-center studies where sharing of individual-level data is not always feasible or preferred. Our method produced the same risk ratio estimates and sandwich variance estimates as the corresponding pooled individual-level data analysis without pooling individual-level data across data partners. The required summary-level information does not contain any potentially identifiable individual-level data and therefore offers better privacy protection. Analytic methods like the one we proposed here complement appropriate governance and data use agreements to enable the conduct of multi-center studies, especially when sharing of individual-level data is challenging.

In terms of privacy protection, the proposed summary-level modified Poisson method serves as an intermediate approach between meta-analysis of site-specific effect estimates from modified Poisson analyses and modified Poisson analysis using pooled individual-level data. Compared to meta-analysis of site-specific effect estimates, our method requires more granular information, but the shared information is summary-level without detailed individual-level data. Unlike meta-analysis that generally only produces approximate results, our method produces results identical to those obtained from the corresponding pooled individual-level data analysis.

Compared to the pooled individual-level analysis, however, our method requires multiple file transfers between the data partners and the analysis center. Although this need for information exchange at each iteration means that our proposed method is more labor-intensive to implement in practice, recent advancements in bioinformatics now allow semi-automated or fully-automated file transfers between data partners and the analysis center [17, 21–27]. For general users who may not have access to such technical infrastructure, we have developed R code that allows manual implementation of our proposed method. This R code (available in Additional file 2) illustrates the analysis of the simulated data from this study but can be easily modified to accommodate different numbers of covariates or different numbers of participating data partners.

We assumed the outcome occurrence between individuals to be independent in our analysis. In some real-world situations, this independence assumption may be violated. In our case, it is possible that individuals who seek care in the same delivery system have correlated outcomes. To account for correlated data, Zou and Donner [28] extended the modified Poisson approach to settings with correlated binary outcomes in single-database studies. Future work will extend the proposed summary-level modified Poisson method to analyze correlated data in multi-center distributed data environments.

## Conclusions

In conclusion, we proposed a privacy-protecting approach to directly estimate adjusted risk ratios using modified Poisson regression analysis for multi-center studies. This approach does not require sharing of individual-level data across data partners but produces results that are identical to those obtained from the corresponding pooled individual-level data analysis.

## Supplementary information

**Additional file 1.** Reports the shared summary-level information in the simulated data example.

**Additional file 2.** Provides replication R code for the data generation and analysis of the simulated data example.

## Data Availability

Replication R code for generating and analyzing the simulated data example is available online and can be easily modified by interested readers for their own multi-center analyses. The real-world data created from the IBM® MarketScan® Research Databases are currently not available for public sharing.
