# Supplementary material for: Privacy-protecting estimation of adjusted risk ratios using modified Poisson regression in multi-center studies
Source: BMC Med Res Methodol. 2019 Dec 5;19:228. doi: 10.1186/s12874-019-0878-6 (PMC6894462; doi:10.1186/s12874-019-0878-6)
Supplement: Supplementary file 1 — Additional file 1. Reports the shared summary-level information in the simulated data example. [file 12874_2019_878_MOESM1_ESM.docx]

**Additional files for**

**“Privacy-Protecting Estimation of Adjusted Risk Ratios Using Modified Poisson Regression in Multi-Center Studies”**

Di Shu*, Jessica G. Young, Sengwee Toh

Department of Population Medicine, Harvard Medical School and Harvard Pilgrim Health Care Institute, Boston MA

[Di_Shu@harvardpilgrim.org](mailto:Di_Shu@harvardpilgrim.org) AND [jyoung@hsph.harvard.edu](mailto:jyoung@hsph.harvard.edu) AND [darren_toh@harvardpilgrim.org](mailto:darren_toh@harvardpilgrim.org)

* Correspondence to Di Shu, Department of Population Medicine, Harvard Medical School and Harvard Pilgrim Health Care Institute, Boston MA (e-mail: Di_Shu@harvardpilgrim.org).

# **Additional file 1**

We fitted a Poisson regression using available R function with pooled individual-level data and obtained the following outputs:

> glm(Y~E+X1+X2+X3+X4+X5,data=data,family=poisson(link=log))$coefficient

(Intercept) E X1 X2 X3 X4 X5

-0.09702882 -0.48776703 -0.38249121 -0.62968463 -0.50382664 -0.09079213 0.13274741

So $\hat{\boldsymbol{\beta}}=(-0.09702882, -0.48776703, -0.38249121, -0.62968463, -0.50382664, -0.09079213,$ $0.13274741)$.

We wrote R code to conduct Newton-Raphson iteration for the proposed summary-level modified Poisson method. The results for each iteration until convergence are given below.

Newton-Raphson Iteration (Results of the proposed summary-level modified Poisson method):

0th iteration:

Set the starting value to be $\boldsymbol{\beta}^{(0)}{=(0,0,0,0,0,0,0)}^{T}$.

1st iteration:

$\boldsymbol{\beta}^{(1)}$=

[1,] -0.36717122

[2,] -0.14009861

[3,] -0.11848422

[4,] -0.18500670

[5,] -0.11057141

[6,] -0.02714062

[7,] 0.04319699

${\boldsymbol{S}_{1}}^{\left( 1 \right)}=$ -3642 -1701 -2354 -1907.324 -3983.257 -3642 0

${\boldsymbol{S}_{2}}^{\left( 1 \right)}=$ -1292 -486 -832 -681.4381 -1521.921 0 -1292

${\boldsymbol{S}_{3}}^{\left( 1 \right)}=$ -2081 -875 -1378 -1088.775 -2326.59 0 0

${\boldsymbol{H}_{1}}^{\left( 1 \right)}$=

[,1] [,2] [,3] [,4] [,5] [,6] [,7]

[1,] -5000.000 -2160.000 -3013.000 -2504.752 -4950.999 -5000.000 0

[2,] -2160.000 -2160.000 -1557.000 -1153.650 -1358.478 -2160.000 0

[3,] -3013.000 -1557.000 -3013.000 -1524.507 -2925.808 -3013.000 0

[4,] -2504.752 -1153.650 -1524.507 -1673.326 -2491.504 -2504.752 0

[5,] -4950.999 -1358.478 -2925.808 -2491.504 -9757.016 -4950.999 0

[6,] -5000.000 -2160.000 -3013.000 -2504.752 -4950.999 -5000.000 0

[7,] 0.000 0.000 0.000 0.000 0.000 0.000 0

${\boldsymbol{H}_{2}}^{\left( 1 \right)}$=

[,1] [,2] [,3] [,4] [,5] [,6] [,7]

[1,] -2000.000 -685.0000 -1176.0000 -995.2110 -2031.5002 0 -2000.000

[2,] -685.000 -685.0000 -481.0000 -377.0464 -391.0787 0 -685.000

[3,] -1176.000 -481.0000 -1176.0000 -583.3638 -1204.9039 0 -1176.000

[4,] -995.211 -377.0464 -583.3638 -656.3050 -1006.1026 0 -995.211

[5,] -2031.500 -391.0787 -1204.9039 -1006.1026 -4078.6824 0 -2031.500

[6,] 0.000 0.0000 0.0000 0.0000 0.0000 0 0.000

[7,] -2000.000 -685.0000 -1176.0000 -995.2110 -2031.5002 0 -2000.000

${\boldsymbol{H}_{3}}^{\left( 1 \right)}$=

[,1] [,2] [,3] [,4] [,5] [,6] [,7]

[1,] -3000.000 -1142.0000 -1825.0000 -1490.9527 -2961.1975 0 0

[2,] -1142.000 -1142.0000 -838.0000 -623.7950 -702.4819 0 0

[3,] -1825.000 -838.0000 -1825.0000 -888.0598 -1793.9868 0 0

[4,] -1490.953 -623.7950 -888.0598 -989.4598 -1468.9650 0 0

[5,] -2961.197 -702.4819 -1793.9868 -1468.9650 -5787.3722 0 0

[6,] 0.000 0.0000 0.0000 0.0000 0.0000 0 0

[7,] 0.000 0.0000 0.0000 0.0000 0.0000 0 0

2nd iteration:

$\boldsymbol{\beta}^{(2)}$=

[1,] -0.30336536

[2,] -0.34085486

[3,] -0.27860864

[4,] -0.44572674

[5,] -0.29650889

[6,] -0.06576835

[7,] 0.09941655

${\boldsymbol{S}_{1}}^{\left( 2 \right)}=$ -1079.556 -528.4264 -724.1273 -579.916 -1260.253 -1079.556 0

${\boldsymbol{S}_{2}}^{\left( 2 \right)}=$ -350.7952 -138.5451 -241.631 -195.0122 -478.3274 0 -350.7952

${\boldsymbol{S}_{3}}^{\left( 2 \right)}=$ -595.2223 -267.9155 -422.5481 -324.3397 -741.1099 0 0

${\boldsymbol{H}_{1}}^{\left( 2 \right)}$=

[,1] [,2] [,3] [,4] [,5] [,6] [,7]

[1,] -2437.5562 -987.4264 -1383.1273 -1177.3435 -2227.995 -2437.5562 0

[2,] -987.4264 -987.4264 -685.1897 -510.9307 -580.960 -987.4264 0

[3,] -1383.1273 -685.1897 -1383.1273 -673.2366 -1238.388 -1383.1273 0

[4,] -1177.3435 -510.9307 -673.2366 -771.9656 -1079.176 -1177.3435 0

[5,] -2227.9953 -580.9600 -1238.3882 -1079.1764 -3987.382 -2227.9953 0

[6,] -2437.5562 -987.4264 -1383.1273 -1177.3435 -2227.995 -2437.5562 0

[7,] 0.0000 0.0000 0.0000 0.0000 0.000 0.0000 0

${\boldsymbol{H}_{2}}^{\left( 2 \right)}$=

[,1] [,2] [,3] [,4] [,5] [,6] [,7]

[1,] -1058.7952 -337.5451 -585.6310 -508.7851 -987.9068 0 -1058.7952

[2,] -337.5451 -337.5451 -228.6170 -181.2181 -181.9676 0 -337.5451

[3,] -585.6310 -228.6170 -585.6310 -280.4111 -550.9613 0 -585.6310

[4,] -508.7851 -181.2181 -280.4111 -329.4848 -472.9413 0 -508.7851

[5,] -987.9068 -181.9676 -550.9613 -472.9413 -1813.9645 0 -987.9068

[6,] 0.0000 0.0000 0.0000 0.0000 0.0000 0 0.0000

[7,] -1058.7952 -337.5451 -585.6310 -508.7851 -987.9068 0 -1058.7952

${\boldsymbol{H}_{3}}^{\left( 2 \right)}$=

[,1] [,2] [,3] [,4] [,5] [,6] [,7]

[1,] -1514.2223 -534.9155 -869.5481 -726.5172 -1375.7174 0 0

[2,] -534.9155 -534.9155 -380.4165 -284.8677 -307.5658 0 0

[3,] -869.5481 -380.4165 -869.5481 -407.7607 -790.2908 0 0

[4,] -726.5172 -284.8677 -407.7607 -474.0610 -660.6156 0 0

[5,] -1375.7174 -307.5658 -790.2908 -660.6156 -2466.2947 0 0

[6,] 0.0000 0.0000 0.0000 0.0000 0.0000 0 0

[7,] 0.0000 0.0000 0.0000 0.0000 0.0000 0 0

3rd iteration:

$\boldsymbol{\beta}^{(3)}$=

[1,] -0.13518535

[2,] -0.46703158

[3,] -0.36958605

[4,] -0.60488845

[5,] -0.45705894

[6,] -0.08814781

[7,] 0.12883735

${\boldsymbol{S}_{1}}^{\left( 3 \right)}=$ -240.6861 -128.4676 -170.5774 -135.2958 -325.041 -240.6861 0

${\boldsymbol{S}_{2}}^{\left( 3 \right)}=$ -68.65836 -22.85332 -48.13311 -40.77635 -125.9112 0 -68.65836

${\boldsymbol{S}_{3}}^{\left( 3 \right)}=$ -125.3072 -62.02884 -102.5445 -73.38744 -198.3736 0 0

${\boldsymbol{H}_{1}}^{\left( 3 \right)}$=

[,1] [,2] [,3] [,4] [,5] [,6] [,7]

[1,] -1598.6861 -587.4676 -829.5774 -732.7232 -1292.7832 -1598.6861 0

[2,] -587.4676 -587.4676 -384.9314 -290.0294 -311.6275 -587.4676 0

[3,] -829.5774 -384.9314 -829.5774 -381.2068 -657.7645 -829.5774 0

[4,] -732.7232 -290.0294 -381.2068 -467.4090 -592.7813 -732.7232 0

[5,] -1292.7832 -311.6275 -657.7645 -592.7813 -2026.4267 -1292.7832 0

[6,] -1598.6861 -587.4676 -829.5774 -732.7232 -1292.7832 -1598.6861 0

[7,] 0.0000 0.0000 0.0000 0.0000 0.0000 0.0000 0

${\boldsymbol{H}_{2}}^{\left( 3 \right)}$=

[,1] [,2] [,3] [,4] [,5] [,6] [,7]

[1,] -776.6584 -221.8533 -392.1331 -354.5493 -635.4906 0 -776.6584

[2,] -221.8533 -221.8533 -142.4919 -114.8564 -109.3339 0 -221.8533

[3,] -392.1331 -142.4919 -392.1331 -178.2977 -323.0714 0 -392.1331

[4,] -354.5493 -114.8564 -178.2977 -223.5560 -289.5395 0 -354.5493

[5,] -635.4906 -109.3339 -323.0714 -289.5395 -1014.8528 0 -635.4906

[6,] 0.0000 0.0000 0.0000 0.0000 0.0000 0 0.0000

[7,] -776.6584 -221.8533 -392.1331 -354.5493 -635.4906 0 -776.6584

${\boldsymbol{H}_{3}}^{\left( 3 \right)}$=

[,1] [,2] [,3] [,4] [,5] [,6] [,7]

[1,] -1044.3072 -329.0288 -549.5445 -475.5649 -832.9811 0 0

[2,] -329.0288 -329.0288 -223.2715 -168.7390 -170.4350 0 0

[3,] -549.5445 -223.2715 -549.5445 -243.9412 -441.6113 0 0

[4,] -475.5649 -168.7390 -243.9412 -302.4626 -380.4731 0 0

[5,] -832.9811 -170.4350 -441.6113 -380.4731 -1309.1345 0 0

[6,] 0.0000 0.0000 0.0000 0.0000 0.0000 0 0

[7,] 0.0000 0.0000 0.0000 0.0000 0.0000 0 0

4th iteration:

$\boldsymbol{\beta}^{(4)}$=

[1,] -0.09830343

[2,] -0.48729388

[3,] -0.38225362

[4,] -0.62917968

[5,] -0.50167323

[6,] -0.09077638

[7,] 0.13265938

${\boldsymbol{S}_{1}}^{\left( 4 \right)}=$ -31.16422 -22.89854 -23.18643 -18.96395 -49.16986 -31.16422 0

${\boldsymbol{S}_{2}}^{\left( 4 \right)}=$ -10.3245 6.875418 1.3354 -3.646295 -19.82538 0 -10.3245

${\boldsymbol{S}_{3}}^{\left( 4 \right)}=$ -16.93988 -8.019526 -18.94716 -10.07516 -37.15179 0 0

${\boldsymbol{H}_{1}}^{\left( 4 \right)}$=

[,1] [,2] [,3] [,4] [,5] [,6] [,7]

[1,] -1389.1642 -481.8985 -682.1864 -616.3914 -1016.9120 -1389.1642 0

[2,] -481.8985 -481.8985 -304.4618 -230.7803 -235.6932 -481.8985 0

[3,] -682.1864 -304.4618 -682.1864 -302.0560 -490.5944 -682.1864 0

[4,] -616.3914 -230.7803 -302.0560 -386.4394 -450.9343 -616.3914 0

[5,] -1016.9120 -235.6932 -490.5944 -450.9343 -1440.6650 -1016.9120 0

[6,] -1389.1642 -481.8985 -682.1864 -616.3914 -1016.9120 -1389.1642 0

[7,] 0.0000 0.0000 0.0000 0.0000 0.0000 0.0000 0

${\boldsymbol{H}_{2}}^{\left( 4 \right)}$=

[,1] [,2] [,3] [,4] [,5] [,6] [,7]

[1,] -718.3245 -192.12458 -342.6646 -317.41923 -529.4047 0 -718.3245

[2,] -192.1246 -192.12458 -119.4698 -97.20874 -88.1431 0 -192.1246

[3,] -342.6646 -119.46984 -342.6646 -150.81298 -253.6824 0 -342.6646

[4,] -317.4192 -97.20874 -150.8130 -196.78155 -233.7902 0 -317.4192

[5,] -529.4047 -88.14310 -253.6824 -233.79018 -757.2551 0 -529.4047

[6,] 0.0000 0.00000 0.0000 0.00000 0.0000 0 0.0000

[7,] -718.3245 -192.12458 -342.6646 -317.41923 -529.4047 0 -718.3245

${\boldsymbol{H}_{3}}^{\left( 4 \right)}$=

[,1] [,2] [,3] [,4] [,5] [,6] [,7]

[1,] -935.9399 -275.0195 -465.9472 -412.2527 -671.7593 0 0

[2,] -275.0195 -275.0195 -181.1554 -137.6167 -131.3510 0 0

[3,] -465.9472 -181.1554 -465.9472 -199.7024 -338.3621 0 0

[4,] -412.2527 -137.6167 -199.7024 -257.8400 -296.8580 0 0

[5,] -671.7593 -131.3510 -338.3621 -296.8580 -951.8083 0 0

[6,] 0.0000 0.0000 0.0000 0.0000 0.0000 0 0

[7,] 0.0000 0.0000 0.0000 0.0000 0.0000 0 0

5th iteration:

$\boldsymbol{\beta}^{(5)}$=

[1,] -0.09703096

[2,] -0.48776649

[3,] -0.38249108

[4,] -0.62968428

[5,] -0.50382221

[6,] -0.09079219

[7,] 0.13274730

${\boldsymbol{S}_{1}}^{\left( 5 \right)}=$ -1.053308 -9.974381 -3.144995 -2.611042 0.06230134 -1.053308 0

${\boldsymbol{S}_{2}}^{\left( 5 \right)}=$ -0.4525363 10.67226 8.868117 2.302101 2.102798 0 -0.4525363

${\boldsymbol{S}_{3}}^{\left( 5 \right)}=$ -0.6457258 -1.250408 -7.000552 -0.7799405 -6.642125 0 0

${\boldsymbol{H}_{1}}^{\left( 5 \right)}$=

[,1] [,2] [,3] [,4] [,5] [,6] [,7]

[1,] -1359.0533 -468.9744 -662.1450 -600.0385 -967.6799 -1359.0533 0

[2,] -468.9744 -468.9744 -294.6183 -223.4654 -224.5974 -468.9744 0

[3,] -662.1450 -294.6183 -662.1450 -291.4107 -463.4464 -662.1450 0

[4,] -600.0385 -223.4654 -291.4107 -375.1789 -426.9297 -600.0385 0

[5,] -967.6799 -224.5974 -463.4464 -426.9297 -1333.9311 -967.6799 0

[6,] -1359.0533 -468.9744 -662.1450 -600.0385 -967.6799 -1359.0533 0

[7,] 0.0000 0.0000 0.0000 0.0000 0.0000 0.0000 0

${\boldsymbol{H}_{2}}^{\left( 5 \right)}$=

[,1] [,2] [,3] [,4] [,5] [,6] [,7]

[1,] -708.4525 -188.32774 -335.1319 -311.47084 -507.47657 0 -708.4525

[2,] -188.3277 -188.32774 -116.5696 -94.94625 -84.77529 0 -188.3277

[3,] -335.1319 -116.56963 -335.1319 -146.76124 -240.88725 0 -335.1319

[4,] -311.4708 -94.94625 -146.7612 -192.58398 -223.01497 0 -311.4708

[5,] -507.4766 -84.77529 -240.8873 -223.01497 -704.90747 0 -507.4766

[6,] 0.0000 0.00000 0.0000 0.00000 0.00000 0 0.0000

[7,] -708.4525 -188.32774 -335.1319 -311.47084 -507.47657 0 -708.4525

${\boldsymbol{H}_{3}}^{\left( 5 \right)}$=

[,1] [,2] [,3] [,4] [,5] [,6] [,7]

[1,] -919.6457 -268.2504 -454.0006 -402.9574 -641.2496 0 0

[2,] -268.2504 -268.2504 -175.8716 -133.6840 -125.4635 0 0

[3,] -454.0006 -175.8716 -454.0006 -193.5060 -320.4877 0 0

[4,] -402.9574 -133.6840 -193.5060 -251.3555 -281.8513 0 0

[5,] -641.2496 -125.4635 -320.4877 -281.8513 -883.6487 0 0

[6,] 0.0000 0.0000 0.0000 0.0000 0.0000 0 0

[7,] 0.0000 0.0000 0.0000 0.0000 0.0000 0 0

6th iteration:

$\boldsymbol{\beta}^{(6)}$=

[1,] -0.09702882

[2,] -0.48776703

[3,] -0.38249121

[4,] -0.62968463

[5,] -0.50382664

[6,] -0.09079213

[7,] 0.13274741

${\boldsymbol{S}_{1}}^{\left( 6 \right)}=$ -0.001848529 -9.67705 -2.53858 -2.084366 2.140329 -0.001848529 0

${\boldsymbol{S}_{2}}^{\left( 6 \right)}=$ -0.0009144189 10.76273 9.138111 2.534111 3.134938 0 -0.0009144189

${\boldsymbol{S}_{3}}^{\left( 6 \right)}=$ -0.001218741 -1.086322 -6.601623 -0.451607 -5.284255 0 0

${\boldsymbol{H}_{1}}^{\left( 6 \right)}$=

[,1] [,2] [,3] [,4] [,5] [,6] [,7]

[1,] -1358.0018 -468.6770 -661.5386 -599.5118 -965.6019 -1358.0018 0

[2,] -468.6770 -468.6770 -294.3923 -223.2961 -224.2343 -468.6770 0

[3,] -661.5386 -294.3923 -661.5386 -291.1012 -462.3994 -661.5386 0

[4,] -599.5118 -223.2961 -291.1012 -374.8285 -425.9683 -599.5118 0

[5,] -965.6019 -224.2343 -462.3994 -425.9683 -1329.2852 -965.6019 0

[6,] -1358.0018 -468.6770 -661.5386 -599.5118 -965.6019 -1358.0018 0

[7,] 0.0000 0.0000 0.0000 0.0000 0.0000 0.0000 0

${\boldsymbol{H}_{2}}^{\left( 6 \right)}$=

[,1] [,2] [,3] [,4] [,5] [,6] [,7]

[1,] -708.0009 -188.23727 -334.8619 -311.23883 -506.44443 0 -708.0009

[2,] -188.2373 -188.23727 -116.5036 -94.89336 -84.65792 0 -188.2373

[3,] -334.8619 -116.50357 -334.8619 -146.62811 -240.34075 0 -334.8619

[4,] -311.2388 -94.89336 -146.6281 -192.42974 -222.53797 0 -311.2388

[5,] -506.4444 -84.65792 -240.3407 -222.53797 -702.47757 0 -506.4444

[6,] 0.0000 0.00000 0.0000 0.00000 0.00000 0 0.0000

[7,] -708.0009 -188.23727 -334.8619 -311.23883 -506.44443 0 -708.0009

${\boldsymbol{H}_{3}}^{\left( 6 \right)}$=

[,1] [,2] [,3] [,4] [,5] [,6] [,7]

[1,] -919.0012 -268.0863 -453.6016 -402.6291 -639.8918 0 0

[2,] -268.0863 -268.0863 -175.7440 -133.5889 -125.2640 0 0

[3,] -453.6016 -175.7440 -453.6016 -193.3124 -319.7568 0 0

[4,] -402.6291 -133.5889 -193.3124 -251.1362 -281.2190 0 0

[5,] -639.8918 -125.2640 -319.7568 -281.2190 -880.5876 0 0

[6,] 0.0000 0.0000 0.0000 0.0000 0.0000 0 0

[7,] 0.0000 0.0000 0.0000 0.0000 0.0000 0 0

7th iteration (model converged):

$\boldsymbol{\beta}^{(7)}$=

[1,] -0.09702882

[2,] -0.48776703

[3,] -0.38249121

[4,] -0.62968463

[5,] -0.50382664

[6,] -0.09079213

[7,] 0.13274741

${\boldsymbol{S}_{1}}^{\left( 7 \right)}=$ -7.377596e-09 -9.676714 -2.537634 -2.083504 2.144434 -7.377596e-09 0

${\boldsymbol{S}_{2}}^{\left( 7 \right)}=$ -3.847939e-09 10.76284 9.138585 2.534538 3.137075 0 -3.847939e-09

${\boldsymbol{S}_{3}}^{\left( 7 \right)}=$ -4.985519e-09 -1.086124 -6.600951 -0.451035 -5.281509 0 0

${\boldsymbol{H}_{1}}^{\left( 7 \right)}$=

[,1] [,2] [,3] [,4] [,5] [,6] [,7]

[1,] -1358.0000 -468.6767 -661.5376 -599.5110 -965.5977 -1358.0000 0

[2,] -468.6767 -468.6767 -294.3920 -223.2959 -224.2337 -468.6767 0

[3,] -661.5376 -294.3920 -661.5376 -291.1007 -462.3974 -661.5376 0

[4,] -599.5110 -223.2959 -291.1007 -374.8280 -425.9664 -599.5110 0

[5,] -965.5977 -224.2337 -462.3974 -425.9664 -1329.2759 -965.5977 0

[6,] -1358.0000 -468.6767 -661.5376 -599.5110 -965.5977 -1358.0000 0

[7,] 0.0000 0.0000 0.0000 0.0000 0.0000 0.0000 0

${\boldsymbol{H}_{2}}^{\left( 7 \right)}$=

[,1] [,2] [,3] [,4] [,5] [,6] [,7]

[1,] -708.0000 -188.23716 -334.8614 -311.2384 -506.44229 0 -708.0000

[2,] -188.2372 -188.23716 -116.5035 -94.8933 -84.65771 0 -188.2372

[3,] -334.8614 -116.50350 -334.8614 -146.6279 -240.33967 0 -334.8614

[4,] -311.2384 -94.89330 -146.6279 -192.4295 -222.53701 0 -311.2384

[5,] -506.4423 -84.65771 -240.3397 -222.5370 -702.47254 0 -506.4423

[6,] 0.0000 0.00000 0.0000 0.0000 0.00000 0 0.0000

[7,] -708.0000 -188.23716 -334.8614 -311.2384 -506.44229 0 -708.0000

${\boldsymbol{H}_{3}}^{\left( 7 \right)}$=

[,1] [,2] [,3] [,4] [,5] [,6] [,7]

[1,] -919.0000 -268.0861 -453.6010 -402.6285 -639.8890 0 0

[2,] -268.0861 -268.0861 -175.7438 -133.5888 -125.2637 0 0

[3,] -453.6010 -175.7438 -453.6010 -193.3121 -319.7554 0 0

[4,] -402.6285 -133.5888 -193.3121 -251.1359 -281.2178 0 0

[5,] -639.8890 -125.2637 -319.7554 -281.2178 -880.5813 0 0

[6,] 0.0000 0.0000 0.0000 0.0000 0.0000 0 0

[7,] 0.0000 0.0000 0.0000 0.0000 0.0000 0 0

Sandwich Variance Estimation:

Using pooled individual-level data, we obtained the sandwich variance estimates below.

[,1] [,2] [,3] [,4] [,5] [,6] [,7]

[1,] 0.0014077439 -1.700711e-04 -2.974435e-04 -1.004883e-03 -3.333412e-04 -6.105324e-04 -6.843484e-04

[2,] -0.0001700711 1.198880e-03 -2.308464e-04 -2.696456e-04 1.696539e-04 -8.910102e-05 3.270397e-05

[3,] -0.0002974435 -2.308464e-04 8.811685e-04 5.400136e-05 -6.074153e-05 8.690003e-06 1.567017e-05

[4,] -0.0010048832 -2.696456e-04 5.400136e-05 2.663668e-03 -5.406249e-05 -1.274835e-05 2.812381e-05

[5,] -0.0003333412 1.696539e-04 -6.074153e-05 -5.406249e-05 5.711052e-04 -3.398284e-05 -1.313090e-06

[6,] -0.0006105324 -8.910102e-05 8.690003e-06 -1.274835e-05 -3.398284e-05 1.148617e-03 6.550177e-04

[7,] -0.0006843484 3.270397e-05 1.567017e-05 2.812381e-05 -1.313090e-06 6.550177e-04 1.454867e-03

Using pooled individual-level data, we obtained the standard errors below.

0.03751991 0.03462485 0.02968448 0.05161073 0.02389781 0.03389126 0.03814272

Using the proposed summary-level modified Poisson method, we obtained the following sandwich variance estimates:

$\hat{var}\left( \hat{\boldsymbol{\beta}} \right)=$

[,1] [,2] [,3] [,4] [,5] [,6] [,7]

[1,] 0.0014077439 -1.700711e-04 -2.974435e-04 -1.004883e-03 -3.333412e-04 -6.105324e-04 -6.843484e-04

[2,] -0.0001700711 1.198880e-03 -2.308464e-04 -2.696456e-04 1.696539e-04 -8.910102e-05 3.270397e-05

[3,] -0.0002974435 -2.308464e-04 8.811685e-04 5.400136e-05 -6.074153e-05 8.690003e-06 1.567017e-05

[4,] -0.0010048832 -2.696456e-04 5.400136e-05 2.663668e-03 -5.406249e-05 -1.274835e-05 2.812381e-05

[5,] -0.0003333412 1.696539e-04 -6.074153e-05 -5.406249e-05 5.711052e-04 -3.398284e-05 -1.313090e-06

[6,] -0.0006105324 -8.910102e-05 8.690003e-06 -1.274835e-05 -3.398284e-05 1.148617e-03 6.550177e-04

[7,] -0.0006843484 3.270397e-05 1.567017e-05 2.812381e-05 -1.313090e-06 6.550177e-04 1.454867e-03

Using the proposed summary-level modified Poisson method, we obtained the following standard errors:

0.03751991 0.03462485 0.02968448 0.05161073 0.02389781 0.03389126 0.03814272

$\boldsymbol{H}_{1}(\hat{\boldsymbol{\beta}})=$

[,1] [,2] [,3] [,4] [,5] [,6] [,7]

[1,] -1358.0000 -468.6767 -661.5376 -599.5110 -965.5977 -1358.0000 0

[2,] -468.6767 -468.6767 -294.3920 -223.2959 -224.2337 -468.6767 0

[3,] -661.5376 -294.3920 -661.5376 -291.1007 -462.3974 -661.5376 0

[4,] -599.5110 -223.2959 -291.1007 -374.8280 -425.9664 -599.5110 0

[5,] -965.5977 -224.2337 -462.3974 -425.9664 -1329.2759 -965.5977 0

[6,] -1358.0000 -468.6767 -661.5376 -599.5110 -965.5977 -1358.0000 0

[7,] 0.0000 0.0000 0.0000 0.0000 0.0000 0.0000 0

$\boldsymbol{H}_{2}(\hat{\boldsymbol{\beta}})=$

[,1] [,2] [,3] [,4] [,5] [,6] [,7]

[1,] -708.0000 -188.23716 -334.8614 -311.2384 -506.44229 0 -708.0000

[2,] -188.2372 -188.23716 -116.5035 -94.8933 -84.65771 0 -188.2372

[3,] -334.8614 -116.50350 -334.8614 -146.6279 -240.33967 0 -334.8614

[4,] -311.2384 -94.89330 -146.6279 -192.4295 -222.53701 0 -311.2384

[5,] -506.4423 -84.65771 -240.3397 -222.5370 -702.47254 0 -506.4423

[6,] 0.0000 0.00000 0.0000 0.0000 0.00000 0 0.0000

[7,] -708.0000 -188.23716 -334.8614 -311.2384 -506.44229 0 -708.0000

$\boldsymbol{H}_{3}(\hat{\boldsymbol{\beta}})=$

[,1] [,2] [,3] [,4] [,5] [,6] [,7]

[1,] -919.0000 -268.0861 -453.6010 -402.6285 -639.8890 0 0

[2,] -268.0861 -268.0861 -175.7438 -133.5888 -125.2637 0 0

[3,] -453.6010 -175.7438 -453.6010 -193.3121 -319.7554 0 0

[4,] -402.6285 -133.5888 -193.3121 -251.1359 -281.2178 0 0

[5,] -639.8890 -125.2637 -319.7554 -281.2178 -880.5813 0 0

[6,] 0.0000 0.0000 0.0000 0.0000 0.0000 0 0

[7,] 0.0000 0.0000 0.0000 0.0000 0.0000 0 0

$\boldsymbol{B}_{1}(\hat{\boldsymbol{\beta}})=$

[,1] [,2] [,3] [,4] [,5] [,6] [,7]

[1,] 893.8094 343.9908 484.8420 414.2163 706.7392 893.8094 0

[2,] 343.9908 343.9908 227.3160 163.8169 173.8047 343.9908 0

[3,] 484.8420 227.3160 484.8420 222.4398 352.7863 484.8420 0

[4,] 414.2163 163.8169 222.4398 265.3111 323.8006 414.2163 0

[5,] 706.7392 173.8047 352.7863 323.8006 1055.2438 706.7392 0

[6,] 893.8094 343.9908 484.8420 414.2163 706.7392 893.8094 0

[7,] 0.0000 0.0000 0.0000 0.0000 0.0000 0.0000 0

$\boldsymbol{B}_{2}(\hat{\boldsymbol{\beta}})=$

[,1] [,2] [,3] [,4] [,5] [,6] [,7]

[1,] 399.2201 138.18055 223.85354 196.2517 340.50865 0 399.2201

[2,] 138.1805 138.18055 91.48663 74.3128 73.85344 0 138.1805

[3,] 223.8535 91.48663 223.85354 107.4831 178.57922 0 223.8535

[4,] 196.2517 74.31280 107.48314 127.1058 163.22134 0 196.2517

[5,] 340.5087 73.85344 178.57922 163.2213 535.34574 0 340.5087

[6,] 0.0000 0.00000 0.00000 0.0000 0.00000 0 0.0000

[7,] 399.2201 138.18055 223.85354 196.2517 340.50865 0 399.2201

$\boldsymbol{B}_{3}(\hat{\boldsymbol{\beta}})=$

[,1] [,2] [,3] [,4] [,5] [,6] [,7]

[1,] 555.0240 197.5328 316.8664 261.5760 451.3865 0 0

[2,] 197.5328 197.5328 136.4933 101.2052 100.5753 0 0

[3,] 316.8664 136.4933 316.8664 141.5665 252.8927 0 0

[4,] 261.5760 101.2052 141.5665 167.8540 209.7546 0 0

[5,] 451.3865 100.5753 252.8927 209.7546 705.5882 0 0

[6,] 0.0000 0.0000 0.0000 0.0000 0.0000 0 0

[7,] 0.0000 0.0000 0.0000 0.0000 0.0000 0 0
