# Supplementary material for: Privacy-protecting estimation of adjusted risk ratios using modified Poisson regression in multi-center studies
Source: BMC Med Res Methodol. 2019 Dec 5;19:228. doi: 10.1186/s12874-019-0878-6 (PMC6894462; doi:10.1186/s12874-019-0878-6)
Supplement: Supplementary file 2 — Additional file 2. Provides replication R code for the data generation and analysis of the simulated data example. [file 12874_2019_878_MOESM2_ESM.docx]

# **Additional file 2**

We provide R code for the simulated data example in the paper. Running the following code in R will reproduce the point estimates and standard errors reported in the paper. Detailed comments are inserted in the R script for readability. In practice, users may have different number of covariates and/or different number of data partners. The code here can be slightly modified for specific questions.

######Data Generation######

#set sample size n

n=10000

#set seed to have reproducible results

set.seed(1000)

#generate covariates X1, X2 and X3

X1= rbinom(n,1,0.6)

X2= runif(n,0,1)

X3= rexp(n)

#generate data source indicators X4 and X5

X4= c(rep(1,n/2), rep(0,n/2))

X5= c(rep(0,n/2),rep(1,n/5), rep(0,3*n/10))

#generate exposure E

E=rbinom(n,1,1/(1+exp(0.73-X1-X2+X3-0.2*X4+0.2*X5)))

#generate outcome Y

Z =-0.1 - 0.4*X1-0.6*X2-0.5*X3 -0.1*X4 +0.1*X5 - 0.5*E

prb = exp(Z)

Y = rbinom(n,1,prb)

#create the pooled individual-level data

data = data.frame(Y=Y,E=E,X1=X1,X2=X2,X3=X3,X4=X4,X5=X5)

#create 3 data partners DP1, DP2 and DP3

DP1=subset(data, X4==1)

DP2=subset(data, X5==1)

DP3=subset(data, (1-X4)*(1-X5)==1)

#pooled individual-level data analysis for point estimation

glm(Y~E+X1+X2+X3+X4+X5,data=data,family=poisson(link=log))$coefficient

#proposed distributed modified Poisson method for point estimation

#write a function "iterate" to process an iteration

#input bb, the value of beta in the previous iteration

#output the value of beta in the current iteration

iterate<-function(bb){

Xmat1=as.matrix(data.frame(1,DP1$E,DP1$X1,DP1$X2,DP1$X3,DP1$X4,DP1$X5))

Xmat2=as.matrix(data.frame(1,DP2$E,DP2$X1,DP2$X2,DP2$X3,DP2$X4,DP2$X5))

Xmat3=as.matrix(data.frame(1,DP3$E,DP3$X1,DP3$X2,DP3$X3,DP3$X4,DP3$X5))

Z1=as.vector(Xmat1%*%bb)

Z2=as.vector(Xmat2%*%bb)

Z3=as.vector(Xmat3%*%bb)

S1=t(Xmat1)%*%(DP1$Y-exp(Z1))

S2=t(Xmat2)%*%(DP2$Y-exp(Z2))

S3=t(Xmat3)%*%(DP3$Y-exp(Z3))

H1=-t(Xmat1)%*%(diag(exp(Z1))%*%Xmat1)

H2=-t(Xmat2)%*%(diag(exp(Z2))%*%Xmat2)

H3=-t(Xmat3)%*%(diag(exp(Z3))%*%Xmat3)

colnames(S1)=NULL

colnames(S2)=NULL

colnames(S3)=NULL

colnames(H1)=NULL

colnames(H2)=NULL

colnames(H3)=NULL

rownames(S1)=NULL

rownames(S2)=NULL

rownames(S3)=NULL

rownames(H1)=NULL

rownames(H2)=NULL

rownames(H3)=NULL

H=H1+H2+H3

S=S1+S2+S3

bb-(solve(H))%*%S

}

#Suppose the initial value is a zero vector

#beta1=iterate(rep(0,7)) gives the beta estimates in the first iteration

#The second iteration: beta2=iterate(beta1)

#The third iteration: beta3=iterate(beta2)

#and so forth

#It took 7 iterations to converge

beta1=iterate(rep(0,7))

beta1

beta2=iterate(beta1)

beta2

beta3=iterate(beta2)

beta3

beta4=iterate(beta3)

beta4

beta5=iterate(beta4)

beta5

beta6=iterate(beta5)

beta6

beta7=iterate(beta6)

beta7

######sandwich variance estimation######

#write a function "sandVarPool" to calculate sandwich variance estimates

#using the pooled individual-level data across sites

#input bb, the beta estimates obtained in modified Poisson regression

#output the sandwich variance estimates

sandVarPool<-function(bb){

Xmat=as.matrix(data.frame(1,E,X1,X2,X3,X4,X5))

Z=as.vector(Xmat%*%bb)

H=-t(Xmat)%*%(diag(exp(Z))%*%Xmat)

B=t(Xmat)%*%(diag((Y-exp(Z))^2)%*%Xmat)

colnames(B)=NULL

colnames(H)=NULL

rownames(B)=NULL

rownames(H)=NULL

Hm=solve(H)

Hm%*%B%*%Hm

}

#calculate the sandwich variance estimates

beta=glm(Y~E+X1+X2+X3+X4+X5,data=data,family=poisson(link=log))$coefficient

sandVarPool(beta)

#calculate the standard errors for all elements of beta

(diag(sandVarPool(beta)))^0.5

#write a function "sandVar" to calculate sandwich variance estimates

#using the proposed distributed modified Poisson method

#input bb, the beta estimates obtained in the final iteration

#output the sandwich variance estimates

sandVar<-function(bb){

Xmat1=as.matrix(data.frame(1,DP1$E,DP1$X1,DP1$X2,DP1$X3,DP1$X4,DP1$X5))

Xmat2=as.matrix(data.frame(1,DP2$E,DP2$X1,DP2$X2,DP2$X3,DP2$X4,DP2$X5))

Xmat3=as.matrix(data.frame(1,DP3$E,DP3$X1,DP3$X2,DP3$X3,DP3$X4,DP3$X5))

Z1=as.vector(Xmat1%*%bb)

Z2=as.vector(Xmat2%*%bb)

Z3=as.vector(Xmat3%*%bb)

H1=-t(Xmat1)%*%(diag(exp(Z1))%*%Xmat1)

H2=-t(Xmat2)%*%(diag(exp(Z2))%*%Xmat2)

H3=-t(Xmat3)%*%(diag(exp(Z3))%*%Xmat3)

B1=t(Xmat1)%*%(diag((DP1$Y-exp(Z1))^2)%*%Xmat1)

B2=t(Xmat2)%*%(diag((DP2$Y-exp(Z2))^2)%*%Xmat2)

B3=t(Xmat3)%*%(diag((DP3$Y-exp(Z3))^2)%*%Xmat3)

colnames(B1)=NULL

colnames(B2)=NULL

colnames(B3)=NULL

colnames(H1)=NULL

colnames(H2)=NULL

colnames(H3)=NULL

rownames(B1)=NULL

rownames(B2)=NULL

rownames(B3)=NULL

rownames(H1)=NULL

rownames(H2)=NULL

rownames(H3)=NULL

H=H1+H2+H3

B=B1+B2+B3

Hm=solve(H)

Hm%*%B%*%Hm

}

#calculate the sandwich variance estimates

sandVar(beta7)

#calculate the standard errors for all elements of beta

(diag(sandVar(beta7)))^0.5
